# Cross-sectorial collaboration on policy-driven rehabilitation care models for persons with neuromuscular diseases: reflections and behavior of community-based health professionals

**DOI:** 10.1186/s12913-022-08557-3

**Published:** 2022-09-17

**Authors:** Charlotte Handberg, Ulla Werlauff

**Affiliations:** 1 National Rehabilitation Center for Neuromuscular Diseases, Kongsvang Allé 23, 8000 Aarhus, Denmark; 2 Department of Public Health, Faculty of Health, Aarhus University, Aarhus, Denmark

**Keywords:** Neuromuscular diseases, Healthcare professionals, Collaboration, Fundamental care, Rehabilitation, Community care, Organization, Coordination¸ policy decisions, Culture

## Abstract

**Background:**

Persons with neuromuscular diseases (NMDs) often experience complex rehabilitation needs due to the disease’s impact on their functioning and progression of their diseases. As a consequence of legislation and “policy power”, community-based health professionals function as gatekeepers to the rehabilitation trajectory for persons with NMDs in a field where the other professionals are the specialists.

**Aim:**

To investigate community-based health professionals’ reflections on and behaviors regarding collaboration with a tertiary rehabilitation hospital in a cross-sectorial rehabilitation care model with the overall aim of providing high quality rehabilitation for persons with NMD.

**Methods:**

The design is qualitative and uses interpretive description methodology and the theoretical lens of Edgar Schein’s three levels of organizational culture and leadership.

An ethnographic fieldwork was conducted from September 1, 2019 to January 30, 2020. Eighty-four community-based health professionals were included and 17 of them were interviewed in four semi-structured focus group interviews (*n* = 10) and seven individual interviews (*n* = 7). In addition, 151 pages of observation data were generated. The study adheres to the COREQ guidelines.

**Results:**

The analysis showed three themes of importance for the collaboration: Policy and legislation navigation represented that collaboration on rehabilitation was affected by legislation as a management tool with “the case” as the core element, and goal dilemmas. Cross-sectorial knowledge exchange promoted collaboration on coordinated and facilitated rehabilitation and knowledge sharing as a firm anchoring. Patient ownership negotiations implied collaboration was influenced by knowledge founded power and gatekeeping as a navigation tool.

**Conclusion:**

Three levels of organizational culture and leadership were identified, and this overall structure guided the community-based health professionals in their work and in the complex organizational landscape of collaboration between disconnected healthcare systems. The findings provided insight into behavior and attitudes and the content and the values held by the professionals collaborating across sectors. Future collaboration in rehabilitation models should be multiprofessional and team based. The findings emphasize that it is imperative that managements and professionals strive to strengthen the structure of the collaborative team spirit because this will ensure well-planned, coordinated, and conducted rehabilitation for persons with NMD and enable and support future cross-sectorial collaboration in this rehabilitation model for these persons.

## Introduction

### Study rationale

Persons living with neuromuscular diseases (NMDs) are highly dependent on specialist rehabilitation services to live an independent everyday life [1–3]. Through the theoretical lens of Edgar Schein’s three levels of organizational culture and leadership [4], this study investigated collaboration in a specialized rehabilitation model for with NMDs in disconnected healthcare systems. It sheds light on the basic underlying assumptions that ultimately drive the collaboration and negotiations between community-based health professionals (CBHPs) and a multiprofessional team at a tertiary specialized rehabilitation hospital.

## Background

### NMD and the need for rehabilitation

The term NMD covers amyotrophic lateral sclerosis (ALS) and several chronic hereditary NMD subtypes, which together form a heterogeneous population in terms of symptoms, functioning, and disease progression [1]. Persons with NMDs often experience complex rehabilitation needs, due to the disease’s impact on their functioning leaving this group of persons particularly exposed to disconnected healthcare systems [5, 6]. This group experiences an increasing need of specialized rehabilitation efforts during a lifespan due to the biopsychosocial complexity and progression of their diseases [7, 8]. Specialized rehabilitation interventions can support persons with NMDs to maintain their level of functioning for a longer period and postpone loss of functioning, hospitalizations, and dependence on help [7–9]. To ensure that these needs are met, the Danish Health Authorities have formulated instructions for neurology specialists that emphasize the need for a cross-sectiorial approach to rehabilitation for persons with NMDs [3].

### Disconnected healthcare settings

Disconnected healthcare systems are common all over the world and are responsible for challenges related to cross-sectorial collaboration between healthcare professionals, where patients can be lost or abandoned in the “gap” between systems [10–12]. In Denmark, healthcare systems are divided into two sectors: a regional sector consisting of hospital services and inpatient specialized treatment and care; a primary care sector representing the local community setting responsible for care after (or during) hospitalization and rehabilitation; and private hospitals, general practitioners, and private physiotherapists [10]. The intention is to enable seamless care across sectors so that all persons who need multidisciplinary rehabilitation receive it in the primary care setting [13, 14]. Politically, the two sectors are governed by different administrations, but health agreements are negotiated and renewed between the primary healthcare system and the other systems to ensure that the cooperation among sectors can run as smoothly as conceivable across sectors [10]. Even though the intention is seamless cooperation, the underlying organizational and structural conditions in a healthcare system run by two different administrations give rise to recurring challenges [15, 16]. The structural challenges entail various IT systems and different referral systems and political managements.

In the primary care sector, the local CBHPs are legislatively in charge of assessing the rehabilitation needs of the persons with NMD, and they authorize the needed services like personal assistance, help, care, and/or assistive devices according to the Social Services Act, the Active Employment Effort Act, and the Danish Health Act [17–19]. Therefore, they have great influence and impact on the services provided for persons with NMD. However, there tends to be a divergence between policy potentials and persons with NMDs’ needs that can hamper collaboration between the disconnected healthcare systems.

A tertiary rehabilitation service (a third “healthcare system”) to facilitate cross-sectorial collaboration in the field of NMD is represented by The National Rehabilitation Center for neuromuscular diseases (RCFM), a national highly specialized private hospital under the National Board of Health [18, 20]. The RCFM operates in accordance with the Health Act section 79, which states that each Regional Council provides hospital treatment to persons residing in its region [13]. The RCFM collaborates with and facilitates and supports the local health and social community services and the regional services in relation to NMDs. The RCFM offers highly specialized advice and knowledge on the rehabilitation of persons with rare NMDs and has registered approximately 3600 persons with NMDs. The healthcare professionals at the RCFM work in multiprofessional teams across the country, either at an outpatient clinic or in the homes of the persons with NMD. The healthcare professionals are nurses, physiotherapists, occupational therapists, psychologist, doctors, and social workers, and they work as cross-sectorial facilitators in close cooperation with persons with NMDs and other professionals in the health, social, and educational sectors who coordinate rehabilitation.

### Rehabilitation a collaborative process across systems

The World Health Organization defines rehabilitation as a process aimed at enabling persons with rehabilitation needs to reach and maintain their optimal physical, sensory, intellectual, psychological, and social functioning. Rehabilitation provides disabled people with the tools they need to attain independence and self-determination [21]. Rehabilitation aims to take account of the person’s situation in its entirely and comprises coordinated, coherent, and knowledge-based measures [21]. Owing to legislation and “policy power”, CBHPs function as gatekeepers for the services that support the rehabilitation trajectory for persons with NMD in a field where other professionals are the specialists. To prevent a gap between the healthcare systems and ensure high quality rehabilitation for persons with NMDs, it is essential to deconstruct and understand the complexities of this healthcare puzzle in a more strategic manner to ensure creative and productive collaboration in the future. Therefore, the aim of this study was to investigate community-based CBHP’s reflections on and behavior regarding collaboration with a tertiary rehabilitation hospital in a cross-sectorial rehabilitation care model with the overall aim of providing high quality rehabilitation for persons with NMD.

## Methods

### Design

The design was qualitative in accordance with interpretive description methodology [22, 23].

Interpretive description draws upon established qualitative research methodologies and represents a theoretical framework that can be used to identify challenges in clinical nursing practice. The methodology is inductive and entails coherent conceptual description and in-depth interpretation of relationships and patterns within the phenomenon being examined to improve practice [22, 23].

To analyze, decipher, and understand the organizational cultural levels among the two collaborating sectors in this study, the theoretical lens of Edgar Schein’s three levels of organizational culture and leadership was applied [4]. Culture content and the values held in different organizations or ontologies are complex. This organizational cultural lens provides a possibility to make sense of this complexity and helps us investigate the structure of a culture and develop a perspective on how to analyze the complex cultural landscape we encounter [4]. Schein’s theory makes it possible to differentiate between observed and experienced artefacts based on the espoused values and the basic underlying assumptions that ultimately drive behavior in cross-sectorial collaboration [4].

### Setting

The study was conducted in the context of the RCFM at the outpatient hospital clinic, the CBHPs’ offices, or at the homes of persons with NMDs throughout the country.

### Sample and participants

From August 1, 2019 to January 30, 2020, the first author recruited participants, and from September 1, 2019 to January 30, 2020, she conducted an ethnographic fieldwork comprising semistructured individual and focus groups interviews and participant observations. The sampling was purposive and consisted of CBHPs as the primary participants; the secondary participants were the healthcare professionals from the RCFM that collaborated with the CBHPs and persons with NMDs [23]. We aimed for maximal representation regarding educational background, years of experience, location (urban/rural), and field of law (health act/social service).

Included were CBHPs who worked with the assessment of the rehabilitation needs of persons with NMDs and who authorized rehabilitation services like personal assistance, help, care, and/or assistive devices according to the Social Services Act, Active Employment Effort Act and Danish Health Act. The healthcare professionals included had to have collaborated with the RCFM within the last 3 months. Healthcare professionals with no knowledge of NMDs and the RCFM were excluded.

To recruit participants for the interviews, the first author contacted by email the managements in the three largest municipalities in Denmark (Copenhagen: 613,000 inhabitants, Aarhus: 340,000, and Odense: 202,000) and one medium sized municipality (Slagelse: 77,000) and asked them to appoint CBHPs who collaborated with the RCFM and met the inclusion criteria. To generate observation data, the first author shadowed the healthcare professionals at the RCFM when they collaborated with the CBHPs at the outpatient clinic at the RCFM or in the homes of the persons with NMDs homes across the whole country.

In all, 84 CBHPs (social workers, physiotherapists, occupational therapists, nurses, social educators, teachers, etc.) were included as the primary participants (Table 1). The secondary participants were 23 healthcare professionals from the RCFM (doctors, nurses, physiotherapists, occupational therapists, psychologists, and social workers) who collaborated with the CBHPs on rehabilitation interventions for 42 persons with NMD and their relatives. Four social workers opted out – two due to lack of knowledge on collaboration with the tertiary hospital and two due to staff shortages.

**Table 1:** Demographic data on participants

	(***n*** = 84) (%)
**Sex**	
Female	80 (95)
Male	4 (5)
**Age**	
30–39	6 (7)
40–49	4 (5)
50–59	6 (7)
> 60	2 (2)
Not known	66 (79)
**Educational status**	
Registered Nurse	18 (21)
Student Nurse	1 (1)
Social and Healthcare Worker	3 (4)
Social Worker	15 (18)
Social Educator	4 (5)
Teacher	4 (5)
Physiotherapist	9 (11)
Occupational Therapist	8 (10)
**Unknown educational background**	
Authority and Care Worker	17 (20)
Disability and Psychiatry Worker	5 (6)

### Data

Data were generated during the fieldwork and consisted of interview data and observation data.

### Interview data

Of the 84 participants who were observed during the fieldwork, 17 CBHPs were interviewed in four semi-structured focus group interview (*n* = 10) and seven semi-structured individual interviews (*n* = 7). The interview guide for all interviews (individual and focus group) related to understand CBHPs’ reflections on and behavior in the collaboration culture revolving around rehabilitation for persons with NMDs. Key questions in the interview guide were: What are your thoughts on what is meaningful for persons with NMDs in their everyday lives? What are your perceptions of collaboration with the field of NMDs? What do you find of value in relation to multiprofessional collaboration on rehabilitation for persons with NMDs? What do you find of value in relation to cross-sectorial collaboration on rehabilitation for persons with NMDs? What are you perception of the cross-sectorial collaboration within the field of NMDs? What is of value when working with persons with NMDs in disconnected healthcare systems? What are your thoughts on collaborating with a tertiary hospital on rehabilitation for persons with NMDs? When do you believe a collaboration on a rehabilitation process for persons with NMDs works the best? How would you describe your collaboration with tertiary hospitals (like the RCFM)? Follow-up questions were related to concerns, challenges, roles, interactions, communication, and cooperation.

All interviews were conducted by the first author either physically at the CBHPs’ offices or online due to the COVID-19 pandemic. The interviews lasted between 26 minutes and 1 hour and 28 minutes. All interviews were recorded and transcribed by a student worker.

### Observation data

Additionally, 151 pages of observation data were generated by the first author. The participant observations [23] consisted of field notes (superficial, descriptive, analytical, and reflective) of informal conversations, observations, interactions, and conversations during collaborative situations with the person with NMD, the CBHPs and the professionals from the RCFM during home visits, meetings in the outpatient clinic at RCFM, at school or hospital meetings, or examinations (in the patients’ homes or in the clinic).

Observations followed an observation guide that revolved around Edgar Schein’s three levels of organizational culture and leadership [4]. Observations focused on 1) artefacts, like visible structures and processes, behavior that can be directly observed; 2) espoused values like ideals, values, strengths, ideologies, and rationales behind behavior (which are not also aligned with behavior and artefacts); and 3) the observations focused on basic underlying assumptions like unconscious fundamental values and feelings, behavior, perceptions, thoughts, and feelings – elements that gave rise to initiative behavior in the collaborations.

In interpretative description methodology, the aim is not an endpoint with an assumption of data saturation, which would imply that all information from the participants had been heard so frequently that it could be anticipated [23]. Instead, our sampling was guided by information power due to a broad study aim, and we acknowledged that it was always unknown what information and insight the next participant would bring [23]. We therefore aimed for patterns and relations and more open-ended conceptualizations, where the ongoing evolution of thinking is expected [23].

### Data analysis

**Fig. 1:**
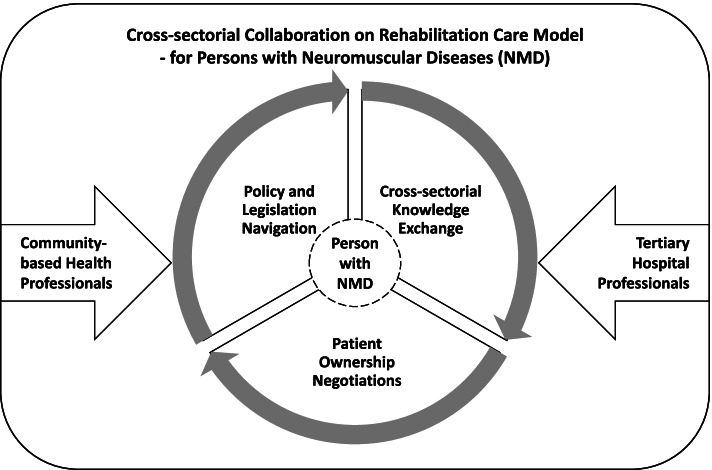
Understanding community-based health professionals’ basic underlying assumptions in regard to collaboration with tertiary rehabilitation hospital professionals in a cross-sectorial rehabilitation care model for persons with neuromuscular diseases

The analysis of all data was conducted by both authors together in an ongoing iterative four-step process according to interpretive methodology [22, 23] (see Table 2). In step 1, all data were transcribed into text and uploaded in NVivo™12, and content coded for each participant and analysis coded with the initial codes. Data were reread and recordings heard again until it was possible to distinguish between special circumstances and generalized patterns in relation to the study purpose [22, 23]. Step 2 consisted of a process that distinguished between special conditions and general patterns in relation to the research aim. Data were analyzed by constant comparison, and themes were derived inductively to identify and determine patterns and relationships [22, 23]. In step 3, a critical assessment was made of the relationships between data, leading to primary categorization and interpretation [22, 23]. Finally, in step 4, the main messages and themes that contributed with new insights related to the study aim were decided upon, and categorical themes determined which formed the final interpretation and thematic structure in relation to the research aim. The hierarchies and relationships of the final themes are illustrated in Fig. 1 [22, 23]. An additional layer of the theoretical lens of Edgar Schein’s three levels of organizational culture and leadership guided the analysis as described above [4].

**Table 2:** Illustration of the analysis and coding process leading up to the final categorical themes guided by the interpretive description methodology (20; 21) and the theoretical lens of Edgar Schein’s three levels of organizational culture and leadership [4]

	First Analytical Step	Second Analytical Step	Third Analytical Step	Fourth Analytical Step
DESCRIPTION OF THE CONTENT AND PROCESS OF THE FOUR ANALYTICAL STEPS IN REGARD TO INTERPRETIVE DESCRIPTION	A process of discernment of particular circumstances and generalized patterns in relation to study aim	A critical appraisal of relationships within data and relevance of thematic options leading to the primary categorization	Extraction of main messages arising from key insights within the data to be captured in the form of a final categorization structure	A model illustrating the hierarchy and relations of the themes and displaying the final findings reported in the findings section.
THEORETICAL LENS OF EDGAR SCHEIN’S THREE LEVELS OF ORGANIZATIONAL CULTURE AND LEADERSHIP	Artifacts (Visual Organizational Structures and Processes)	Exposed Values (Strategies, Goals, Philosophies, Espoused Justifications)	Basic Underlying Assumptions (Unconscious, Taken for Granted Beliefs, Perceptions, Thoughts, and Feelings – Ultimate Source of Values and Action)	
CODES AND SUBTHEMES LEADING UP TO THE FINAL CATAGORICAL THEMES	Legislation as Management Tool Care as Navigation Tool The Case as the Core Element Dilemmas in Collaboration Divergence in the Rehabilitation Understanding	Legislation as Management Tool The Case as the Core Element Rehabilitation Goal Dilemmas	**Policy and Legislation** **Navigation**	
	Coordination and Facilitation of Rehabilitation Knowledge Sharing as a firm Anchoring The Meaning of Tertiary Rehabilitation Collaboration on Several Levels The Meaning of Relations	Coordination and Facilitating Rehabilitation Knowledge Sharing as a firm Anchoring	**Cross-sectorial** **Knowledge Exchange**	
	Knowledge Founded Power Patient Ownership Negotiations The Patients’ Comprehension The Healthcare System as the Gatekeeper	Knowledge Founded Power Gatekeeping as a Navigation Tool	**Patient Ownership** **Negotiations**

## Results

In all, 84 CBHPs participated, nurses and social workers representing the largest groups (Table 1). All had been working in the local community healthcare setting for more than a year and all knew of and collaborated with the RCFM.

The findings provided an understanding of the CBHPs’ basic underlying assumptions in regard to collaboration with tertiary rehabilitation hospital professionals in a cross-sectorial rehabilitation care model for persons with NMDs (Fig. 1). Through the analytical lens, it was possible to understand the overall structure guiding the CBHPs in their work and the complex organizational landscape of collaboration in a shared “territory” in disconnected healthcare systems. The analysis further provided insight into the content and the values held by the CBHPs when collaborating with the professionals at the tertiary hospital. Three categorical themes representing seven subthemes of importance in relation to understanding the content of the collaboration were illustrated, in artefacts and espoused values, but also in the basic underlying assumptions that drove behavior in the collaboration between the professionals in the two organizations (Table 2). Policy and legislation navigation represented collaboration on rehabilitation was affected by having to work with legislation as a management tool, the case as the core element, and the existence of rehabilitation goal dilemmas. Cross-sectorial knowledge exchange promoted collaboration on coordination and facilitated rehabilitation and knowledge sharing as a firm anchoring. Patient ownership negotiations hampered collaboration and were influenced by knowledge founded power and gatekeeping as a navigation tool. Together the three categorical themes intercorrelate and intwine, representing facilitators and barriers to the overall collaboration, which will be further elaborated on in the following.

### Policy and legislation navigation

#### Legislation as management tool

CBHPs used reference to policies and legislations as their management tools during interviews and observations, and thus governance was a core element in their work and collaboration with the hospital professionals. It could be observed how they repeatedly called attention to what “the law” allowed but they mainly referred to what its limits were. The CBHPs tended to talk mostly in terms of legislation, when collaborating, and referred to paragraphs and sections in the law and what these meant with regard to their possibility to provide rehabilitation services.

During a meeting in the home of a woman with NMD, I observed that the CBHP (Female Social Worker, No. 185) discussed needs for rehabilitation initiatives with the person with NMD and the professional from the tertiary hospital. She spoke directly to the person with NMD who had applied for a wheelchair saying: “Typically a wheelchair is used within the home when the disease has progressed more than yours has. When you apply for a wheelchair, it has to be made clear that it is an obvious need and would provide significant relief during your everyday life”. Turning to the professional from the hospital, she referred to the specific law on this subject.

In collaboration at meetings or examinations in the homes of the persons with NMD, it was frequently observed and overheard how the CBHPs used laws and amendments as a lever in their argumentation for postponement or refusal of rehabilitation services. The CBHPs specifically pointed to the fact that policies and legislations made their work easier in some ways. Conversely, some of the participants argued that governance made their work somewhat more difficult and rigid because it left no room for trying out different solutions. In relation to this, some of the participants explained that at times they wished that they were not as bound by governance as they were and instead could provide the rehabilitation initiatives needed by the persons with NMD. However, in their collaboration with the hospital professionals, their strongest argument appeared to be that they could obviously only provide services that they had legal authority to provide.

“It tends to create challenges if the hospital professionals go ahead and suggest rehabilitation interventions or solutions – and the person with NMD applies for them. Then we must decide whether to authorize the solution, and when we reject it, the persons with NMD often gets very upset with us. I think these events are unfortunate and that the professionals at the tertiary hospital need to be as updated on the policies and legislations as possible – they change constantly. The principles on which decisions are based often change. They do”. (Female Occupational Therapist, No. 139).

#### The case as the core element

The CBHPs explained their opinions in more detail during interviews, and it was also observed that “the case” was put before the person. This CBHPs explained that their behavior was rooted in a “legislation culture” rather than in the person with NMD in need of rehabilitation, sometimes with consequences for the outcome of the specific case. The CBHPs explained that they were not willing to apply for assistive aid if they felt that aids did not make sense in relation to governance, for instance, they rejected applications for extensive renovations of houses because of the costs incurred. In such “cases”, they could also argue that the hospital professionals at times showed up too late to collaborate or to provide the written neuromuscular report that was used to strengthen some applications. They further explained that they did not always wait for the hospital professionals to show up, but acted on the knowledge they had at the time, which was mainly concerned with legislation and not facilitated by specific knowledge of the NMDs.

“Of course, we must make ourselves acquainted with what sort of “thing” the specific diagnosis is because it does matter if it is ALS or another diagnosis. So, of course, we do study the diagnoses the persons have. But the diagnosis itself does not “release” a service as such. That comes down to the functioning of the person”. (Female Social Worker, No. 43).

Several of the participants described that being proactive regarding their executive duties and applying for assistive devices before they were actually needed was exceptionally important with ALS due to the rapid progression of the disease. If need be, they acted based on the available information and knowledge, knowing full well that more information and knowledge would provide them with better qualifications to act.

A discussion was heard between two CBHPs regarding the written report from the tertiary hospital as a management tool during “case work”. They explained that they copy-pasted certain text elements from the report to build stronger written arguments in case documents when applying for rehabilitation services like assisted help or personal care. (Female Occupational Therapists, No. 25 and 26).

The CBHPs explained how lack of specific diagnosis-related knowledge tended to affect the case work and authorizations for rehabilitation services negatively – underscoring the importance of access to the knowledge possessed by the professionals from the tertiary hospital.

#### Rehabilitation goal dilemmas

It was heard during the interviews and observed that the exact goal of the rehabilitation initiative shifted in focus and at times seemed indistinct or blurry – not only to the professionals, who each had their own goals, but also to the person with NMD, who at times verbalized yet another goal. The unclear overall aim of the rehabilitation plan seemed to present dilemmas during the observed interactions in the collaborations. The persons with NMD seemed to focus on a wish to solve challenges in everyday life, the hospital professionals had an eye for the diagnosis-specific challenges often related to enhancing functioning, whereas the CBHPs focused on legislation and laws.

During a meeting at the home of a man with a NMD, it was observed that the professionals from the hospital sought to discuss diagnosis-related challenges, whereas the CBHPs took the law and governance as a starting point. The CBHP had many questions related to the arrangement of the bathing situation for the person with NMD in her assessment of the need for an authorization for a remodeling of the bathroom. She observed the man with NMD in various situations in the bathroom and discussed issues related to solving the case in relation to the possibilities within the legislation. However, the person with NMD mentioned other worries than the bathroom. He was worried about being home alone because of the risk of falling. The hospital professionals were talking about a ramp for the wheelchair to get in and out of the house. As the meeting progressed, the three parts kept arguing in different directions. Finally, the CBHP, disregarding everything else going on, concluded that she thought the bathroom could be remodeled within the possibilities of the legislation. (Female Social Worker, No. 115).

At times, the CBHPs argued that the collaboration with the tertiary hospital worked well despite different cultures and goals, and it appeared satisfactory regarding the solutions reached during meetings because all those present had a contribution to make. There seemed to be an uncertainty, however, related to what the persons with NMD specifically needed, which might relate to the lack of shared goalsetting. The CBHPs stated that these uncertainties could be related to different persons being responsible for these “NMD cases” in the local community care setting, which, as a consequence, meant that the competences and knowledge on NMD was at risk of being watered down, with a negative outcome for the persons with NMD.

“Concurrently, we address NMD during our meetings because it is so important to be upfront with the different initiatives and how things progress in these cases. We cannot just sit and wait like in other cases. We do have a huge workload, and this might have an influence on some of the cases that need prioritization, and we do need a little knowledge on this specific group, but we don’t have it. No one has been appointed especially to be responsible for this task.” (Female Registered Nurse, No. 149).

### Cross-sectorial knowledge exchange

#### Coordination and facilitating rehabilitation

The professionals in both healthcare systems contributed with the specific knowledge they possessed to ensure the best possible support and rehabilitation plan for the persons with NMD. The CBHPs argued how important the cross-sectorial collaboration with the tertiary hospital was for the rehabilitation to be coordinated and facilitated well. Often the hospital professionals were responsible for setting up the meetings and gathering the relevant people to participate based on their specific competences. However, the CBHPs explained how they were the “local” and nearest professionals and that, therefore, they were in a better position to follow-up on and execute plans that had been decided upon. Nevertheless, they explained that the facilitating and coordinating role was enforced by a culture of working together with the hospital professionals who had knowledge about the specific NMD diagnoses and related rehabilitation needs. One CBHP said:

“That's what is so good about it. Take the case where I participated. We had a network meeting in the home of the person with NMD – you know there were people representing the local community care setting, from the public day-care facilities, and from what we call the children’s therapy who are our therapists. In addition, our occupational therapist participated; she works with housing adjustments and stuff like that. I participated and then there were professionals from the tertiary hospital, and people from special counseling who had contributed to assessing the boy. In that way we all got to see each other, get acquainted with each other, and get a good discussion going, all of us. In that way we were able to make a shared rehabilitation plan … you know where we go from here. Making sure that we all worked in the same direction so to speak – all professional experts no matter where you work”. (Female Social Worker, No. 147).

The CBHPs mentioned these collaborative meetings several times and the value of being together physically – all in the same room. Moreover, they emphasized how much easier things progressed and were coordinated in a positive manner once you knew the other professionals and understood what they represented. Amidst examinations, this shared facilitation and coordination of rehabilitation initiatives was also observed repeatedly and acknowledged by the persons with NMD and their relatives.

In the process of a network meeting in the house of a girl with NMD, it was observed how a plan was coordinated in collaboration. The girl needed a new wheelchair, and the professionals from the tertiary hospital offered to pass the information on to a colleague who was a specialist occupational therapist who could help with the adjustments of the new wheelchair but also could help make it possible for the girl to drive around on the school premises. The teacher (No. 6) from the school talked about the accessibility at the school in general and classroom placement. The girl’s mother contributed with knowledge and needs regarding the new chair. The hospital physiotherapist offered to coordinate help in the future and informed the mother about who to contact in the local community care setting.

#### Knowledge sharing as a firm anchoring

Knowledge sharing was described as a core element in the collaboration between the professionals in both healthcare systems. The CBHPs possessed specific knowledge on possibilities and/or obstructions in relation to governance, policies, and the specific laws. The hospital professionals had specialist knowledge about the NMDs and the needs of the persons with specific diagnoses as well as the relevant legislation. The two groups adhered to some of the same legislation but used it differently, with the CBHPs being managed by the community care setting’s financial framework in relation to what services they authorized. The two group of professionals explained they benefitted from a culture of collaborating and sharing their specific knowledge with the shared overall aim of helping persons with NMD in the best possible way. The CBHPs described in detail how they used the hospital professionals’ specialized knowledge of NMD to understand the disease and the related needs better. Their better understandings of the detailed needs related to the precise diagnosis strengthening the CBHPs’ arguments in cases and applications for rehabilitation initiatives. The CBHPs revealed that they valued the knowledge sharing during the collaboration they had with the professionals at the hospital.

The CBHP (Female Physiotherapist, No. 168) was asking the hospital physiotherapist about how to interpret a physical score that the physiotherapist used during examinations. They discussed the score, and the hospital physiotherapist explained how the specific NMD affects the body and muscles, and she explained about relevant exercises for the physiotherapist to do together with the person with NMD. The physiotherapist demonstrated the exercises on the boy who was being examined. The boy had spasms during the exercises and the two professionals carried on discussing how his muscles reacted and about muscle power and which exercises to do and which to avoid. The hospital physiotherapist added that she would share the discussed knowledge in the written report she would forward after the meeting.

When sharing knowledge, the collaboration at times seemed to work so well that the professionals could get carried away and had long professional discussions on NMD symptoms and needs. At times, issues were discussed and decisions made over the head of the person with NMD and their relatives (even though present), and the communication ended up being “a case discussion” between the professionals while the persons with NMD listened.

Overall, the CBHPs explained that the exchange of specialized knowledge on NMD was invaluable “gold” and that they were heavily dependent on it in their case work. Nevertheless, The CBHPs also emphasized the value of contributing with their knowledge to help the person with NMD in the best way and the value of the synergy in exchanging information and knowledge.

“They came from the hospital last winter and gave a lecture on ALS. That was so worthwhile for us. It was one of the nurses and in such ways we could do even more to collaborate on knowledge sharing between us. We provide each other with valuable knowledge. They nourish us with knowledge on what is going on within the field of disability “outside” the jurisdiction, and about the newest research and evidence on NMD. At the same time, we can contribute with what is going on regarding governance and legislations and what challenges we encounter in the local community care setting. In that way we can guide each other and together upgrade the work in the field of rehabilitation”. (Female, Social Worker, No. 42).

### Patient ownership negotiations

#### Knowledge founded power

Although the CBHPs explained during interviews that collaboration with the tertiary hospital professionals was seamless, the observations revealed in more detail how challenges occurred. The interviews and the observations provided insight into an ongoing discourse on the “ownership” of the person with NMD, like who knew the person best or who could decide in what direction things should progress in relation to rehabilitation solutions. The CBHPs referred to the person with NMD as “my patient”, as did the professionals at the hospital. During meetings, specific knowledge of the persons with NMD and in addition specialized knowledge – on either legislation for the CBHPs or detailed knowledge of the disease for the hospital professionals – were used as reinforcements when trying to win an argument.

Throughout a school meeting there seemed to be an ongoing fight for power founded in knowledge. The professionals tried to work out rehabilitation initiatives for a teenage girl with NMD, but it was observed recurringly how this was challenged by the professionals from the community-based care setting. A female teacher (No. 154) and a male social educator, (No. 153) participated in the meeting at the girl’s school with a physiotherapist and an occupational therapist from the tertiary hospital. The hospital professionals informed about a prior meeting the same day in the home of the girl with NMD and elaborated on the symptoms of the NMD like falling over, fatigue, spasms, and difficulties with riding a bike. The physiotherapist explained the related needs for the girl during school hours and addressed how these could be supported and relieved. The teacher (No. 154) seemed either uninterested or pointed out how things were during school hours and how they chose to support the girl. The social educator (No. 153) seemed more interested and sought consensus in the group; however, it appeared difficult for him since the teacher kept either demonstratively reading something on her computer or suddenly, when possibilities for the girl with NDM to have a quiet moment were addressed, adding things like: “Our strategy for all classes is to have only a few students in the classroom, keep the noise level low – that’s our strategy in all classrooms, not just the one class room where XX is.”

As shown in the above, the collaboration could, at times, be hindered by the participation of hospital professionals in only one or two meetings and by their not being a part of the local community care setting surrounding the persons with NMD. During some collaboration situations, the CBHPs resented being told “what to do” or “how to act” by the hospital professionals and this led to a feeling of not being good or sufficient enough, which seemed to ruffle their professional pride. The CBHPs argued that they acknowledged the specialized knowledge that the hospital professionals had, but that it was their patients and their decisions that determined what rehabilitation initiatives were authorized and they hinted that also knew the patients best.

“If you take that little boy with NMD we visited and assessed in his home, where we discussed if a case involving remodeling the house was relevant now or later … In that case I sort of found myself in a dilemma, since the parents had been told [by the hospital professionals] to expect and anticipate that a renovation of their house would be authorized. But I wondered if it was necessary and things like that. We had a good discussion that we [the CBHPs] in the end won because we possess the authority power to decide what aid is given and possess the competences to make the decisions. We have the decision-making power to say – we’ll look at it again within a foreseeable period, it will happen eventually”. (Female Occupational Therapist, No. 161).

#### Gatekeeping as a navigation tool

In the quest to show ownership of the patients, the CBHPs emphasized how they were the ones in charge of what was being authorized according to the legislation. The CBHPs explained that, as time progressed, they had acquired more experience and knowledge about NMD, and they could, therefore, make well-informed decisions on the cases they “owned” without necessarily having to collaborate with the tertiary hospital around them. During observations, it became more evident how the CHBPs used their authority and legal framework as a gatekeeping tool.

During a meeting in the home of a couple where the man had ALS, a nurse from the hospital and a CBHP professional participated. The CBHP (Female Nurse, No. 157) introduced herself and explained that she represented the local community care authorities regarding everything relating to personal care and assistance and she then added: “You get the help that you need according to the Social Services Act”. She shared a pamphlet with information regarding possibilities for rehabilitation according to the legislation. She further mentioned that it would be easier for her to authorize rehabilitation services in the future because she had now seen the couples’ house. “I think that it would be a good idea for you to become affiliated with the local home care nurse as soon as possible”. The couple nodded agreement.

The professionals from the tertiary hospital used their knowledge on legislation to inform the patient about possibilities such as those available through the CBHPs. The CBHPs, however, also made it clear that the local community care setting oversaw the provision of services for the person with NMD. The CBHPs emphasizing the importance of being involved in the case at an early stage because they knew what would be needed and could either authorize help or help the persons with NMD navigate in the system so that they could seek out the best help. These clearly mapped “boarders” of who “owned” the responsibility or the possibilities for providing rehabilitation services appeared to promote the development of two parallel healthcare systems – instead of a collaboration. Moreover, this division blurred the shared goal of providing the best possible help and rehabilitation for the persons with NMD.

Nevertheless, the CBHPs stressed that they needed and trusted the hospital professionals’ expertise but that they did not like being pressured to make decisions or authorize services that they did not find appropriate or needed. The CBHPs described that they sometimes felt that the tertiary hospital “ganged up” with the person with NMD and their families, for instance, “promising” them services without legal grounding. In such cases, the CBHPs revealed that they tended to feel like outsiders when they took part in meetings, despite representing the local instance responsible for the help and support needed by the person with NMD.

“The whole point of my position is to function as a navigator for the person with NMD, and sort of get to know the family and sort out what challenges they encounter, what they consist of, and what rehabilitation initiatives are needed, and to facilitate their journey forward to the different actors and departments in the local community care setting. I don’t have authoritative power in my position as such, but I function as the extended arm of the person with NMD, you could say … and I make everyday life easier to cope with.” (Female Social and Healthcare Worker No. 129).

## Discussion

Our findings showed that the culture and values in the two healthcare organizations were complex and challenged during collaboration on rehabilitation for persons with NMDs [4]. We identified three overall elements that influenced collaboration between the CBHPs in the local community-based healthcare setting and the tertiary rehabilitation hospital professionals in the rehabilitation model: navigation dictated by policies and legislation, ongoing cross-sectorial knowledge exchange, and negotiations for patient ownership. The structures in the collaboration in a rehabilitation model (i.e., the Social Services Act, the Active Employment Effort Act, and the Danish Health Act) were, however, interpreted differently by the professionals who collaborated in cross-sectorial settings in the two healthcare systems [4, 17–19]. We found that policy and legislation navigation and patient ownership negotiations challenged and hampered the facilitation of a seamless collaboration between the cross-sectorial teams, which consisted of two smaller teams representing each of the two healthcare systems. Differences appeared engrained in espoused values like the organizational references made by the CBHPs such as “in our organization we do not authorize without reference to the legislation”, and more importantly in basic underlying assumptions “who are we doing this for – what do we base our decisions on (the patients or the law)?”. The CBHPs behaved based on values like managing according to legislation and sharing knowledge founded in legislation; however, they used a claimed “patient ownership” to win arguments. The hospital professionals also claimed patient ownership, and their arguments were also founded in legislation (the Danish Health Act), but their understanding of the legislation was based in an ontological culture where treatment, care, rehabilitation, and palliation are core elements and management tools.

Core elements in rehabilitation teamwork among organizations consist of shared commitment; an understanding of and meaning given to the collaborating team by other healthcare professionals; shared and explicit rehabilitation goals, agreed roles and responsibilities. This delineates the unique and shared areas of authority within the team and an interdependence between team members when making decisions and when undertaking rehabilitation initiatives [24]. Lack of distinct definitions of the structures and elements in the collaborative rehabilitation model in the present study may have presented challenges for the professionals and the organizations. The diverse culture and ontological values among the professionals in the two teams (healthcare systems) seemed at times to clash, which did not always benefit rehabilitation outcomes for the patients with NMD. This clash between teams might be because the goals were not always voiced and clarified by all in the two teams.

Rehabilitation is a complex process, and a shared goal set together with the persons with NMD should be a core component in all collaboration among the healthcare professionals [25].

The general intent of the various legislations used as management tools in the two healthcare settings in our study is to promote the patients’ best interests; hence, it is important that their content and structure are clear to the professionals in each sector and enable them to be certain about their roles and expectations in the collaboration. With regard to arguments and interprofessional power, governance and legislations can become the center of the collaboration and be expressed as strong arguments based on care and health, as was the case with the hospital professionals in the present study. A contextual understanding of the identified elements influencing collaboration in the rehabilitation model for persons with NMDs becomes crucial for the outcome of the rehabilitation and for understanding the content and structures affecting the whole rehabilitation process.

A way to make structures in the collaboration more visible and central in the collaboration process among the professionals could be the use of the Chronic Care Model (CCM). The CCM has throughout more than a decade been a commonly adopted approach to improve care initiatives, and evidence supports the use of the CCM to guide practice changes [26]. A Cochrane collaborative review on the CCM and interventions to improve care has shown that changes in multicomponent practice in four areas can lead to improvements in health outcomes: 1) increase professionals’ expertise and skill, 2) educate and support patients, 3) encourage team-based delivery and planned care, and 4) make increased use of registry-based information systems [26]. Our findings touch upon the first three of these four areas; however, it is imperative to acknowledged that teamwork is an essential structure in the collaboration between rehabilitation teams because healthcare systems are complex and every team represents a piece of the healthcare system [24]. Rehabilitation teams consist of networks, each of which influences the other networks. In our study, the two healthcare systems are also a part of a larger network contributing to the collaborative rehabilitation of persons with NMD. Persons living with NMDs are highly dependent on specialist rehabilitation services, but as shown in the present study, disconnected healthcare systems present challenges in the collaboration between healthcare professionals. Rehabilitation depends on a thorough analysis of the biopsychosocial challenges encountered by the person with NMD and is an initiative that requires collaborative work [24]. The professionals involved must reach a shared and agreed understanding of what the challenges for the specific person with NMD are, what elements are the most important, what is the prognosis, and, most importantly, what interventions and plan will resolve or reduce the difficulties for the person [24]. A combination of the elements mentioned above and those in the CCM might help form a more fixed structure for a cross-sectorial collaborative rehabilitation model for persons with NMDs [26]. Our findings indicate that some of the suggested changes in the CCM already existed in the collaboration among the two teams in the present study, but perhaps were not given sufficient attention.

Teamwork is effective and essential for rehabilitation, and in our study two teams (or healthcare systems) collaborated in one larger team. Rehabilitation teams usually consist of professionals from many different fields to ensure a broad range of competencies to meet the potential challenges a person with NMD may encounter [24]. Research has shown how collaboration and leadership can be critical to the effectiveness of rehabilitation nursing practice and how careful and collaborative rehabilitation initiatives can change the context and culture of care for the better [27]. Likewise, specific structures for rehabilitation management within the field of NMDs have been described as pointing toward the importance of concurrent multidisciplinary team efforts [7, 8]. Finally, investment in co-creation of care (in our study rehabilitation) has been shown to be important for the well-being and job satisfaction of community healthcare nurses [28]. These findings underscore why organizations like the two in our study should pay attention to team efforts across sectors. Nevertheless, findings in a study of a multi-site arts-based intervention to improve patient-centered neurorehabilitation practice have shown that interprofessional collaboration in neurorehabilitation nursing is difficult [29]. These findings emphasized the importance of the intraprofessional collegialism that can be achieved through task and knowledge sharing and emotional support [29]. Fortunately, knowledge exchange was the least problematic area in our findings. Knowledge exchange between the two healthcare systems in our study seemed to work well and helped to form stronger connections among the professionals.

The rehabilitation teams in cross-sectorial settings should make an effort to clearly define roles and allocation of responsibilities to increase trust, openness, respect, and equality [24]. This is especially needed with regard to rehabilitation capabilities, where it must be possible to form a capable and well-working team defined by its reciprocal behavior even though working from a different base like within our study in different health systems. Effective and well-collaborating teams can be characterized by, among other things, a sharing documentation and information and of a common language with terms understood by all team members. Moreover such teams are characterized by undertaking initiatives with the persons with NMD that are usually undertaken by someone from another profession, sharing knowledge and skills, and making their unique expertise available to each other, so that team members can learn from other professions and share responsibilities [24]. Many of these elements were present in the collaboration among the teams in our study, but an enhanced focus by managements and the professionals on these elements will promote a thoughtful and motivated collaboration with the persons with NMD to facilitate meaningful rehabilitation solutions.

### Methodological considerations

Our study sample included various professionals with different backgrounds representing various areas of legislation and governance and was large for a qualitative study. Even though it could be argued that this broad sampling might have influenced the findings by thinning them out, we consider the broad representation a strength because we were looking for trends and patterns in the field of collaboration among the two healthcare systems more than the obtaining of in-depth knowledge from only a few individual participants. To enhance credibility and trustworthiness in the study, several measures were undertaken. Before the onset of the study a thorough explorative process with involvement of six persons with NMD and their relatives was carried out, and all were engaged in commenting on and developing the project protocol regarding relevance, aims, methods, ethics, and perspectives. Based on feedback from these six persons, the perspective of the project was broadened to include persons with ALS, which had not been the initial plan.

The first author was not known by the participants before the study and was at the time of the fieldwork new in the field of NMD, which may have reduced any preunderstanding and enhanced an objective data generation. The second author, however, had many years of experience within the field of NMD, which strengthened our team in relation to posing pertinent interview questions and making salient observations. Moreover, the interview and observation guide were followed during the fieldwork to ensure the cogency and unification of the data [22, 23]. The analysis process was conducted by both authors, with the initial readings and analysis being conducted separately to be able to compare the inductive findings and look for patterns. As the analysis progressed, the findings were discussed concurrently in accordance with the interpretive description methodology of the iterative four-step analysis process (Table 2) [22, 23]. Our findings should be transferable to other similar healthcare settings delivering rehabilitation in relation to a specific cross-sectorial organization model. The Danish healthcare system has some unique features, but our findings are constructed around collaboration between disconnected systems, which we consider will be applicable to many other health systems, contexts, and countries. Future research should investigate the experience of persons with NMD regarding collaboration with healthcare professionals.

## Conclusion

The present study provided insight into the reflections and behavior of CBHPs in their collaboration with the professionals at a tertiary hospital. Our findings illustrated the artefacts and espoused values but also the basic underlying assumptions that drove the behavior in the collaboration among the professionals in the two organizations. Through the analytical lens of Edgar Schein’s three levels of organizational culture and leadership, we identified an overall structure guiding the CBHPs in their work and the complex organizational landscape of collaboration between disconnected healthcare systems. Policy and legislation navigation represented collaboration on rehabilitation as it was affected by having to work with legislation as a management tool, with “the case” as the core element and goal dilemmas. Cross-sectorial knowledge exchange promoted collaboration on coordinated and facilitated rehabilitation and knowledge sharing as an anchoring. Patient ownership negotiations hampered collaboration and were influenced by care negotiations founded in knowledge that was either legislation bound or founded in knowledge on NMDs. Together the three themes intercorrelated and intwined, representing facilitators and barriers to seamless cross-sectional collaboration between healthcare systems. The best solution for optimal management of such complex rehabilitation challenges could be to keep striving for smoothly working collaborative multiprofessional teams of professionals leading toward the agreed common goals. Therefore, managerial focus should be on strengthening the multiprofessional team spirit among the teams working across sectors and setting goals together with the persons with NMD to ensure that everyone is working in the same direction. Finally, it is important that managements and professionals strive to strengthen the collaborative team spirit to enhance benefits for persons with NMDs and ensure well-planned, coordinated, and conducted rehabilitation that will enable and support future cross-sectorial collaboration in rehabilitation models for persons with NMD.

## Data Availability

The data that support the findings of this study are available from Charlotte Handberg, but restrictions apply to the availability of these data, which were used under license for the current study, and so are not publicly available. Data are however available from the authors upon reasonable request and with permission of Charlotte Handberg.
